# Caffeine Intoxication Due to Antipyretic Analgesic Overdose in an Adolescent

**DOI:** 10.7759/cureus.17922

**Published:** 2021-09-13

**Authors:** Yohei Horikawa, Shuichi Yatsuga, Yuki Okamatsu

**Affiliations:** 1 Department of Pediatrics, Aso Iizuka Hospital, Iizuka City, JPN

**Keywords:** caffeine, intoxication, antipyretic analgesic, overdose, suicide

## Abstract

Recently, high concentrations of caffeine present in energy drinks and over-the-counter (OTC) drugs have become a concern worldwide. Several deaths due to caffeine intoxication have been reported, necessitating caution. Typically, supportive care is used to treat caffeine intoxication. However, in severe cases of caffeine intoxication, hemodialysis may be used. For adults, a lethal blood caffeine concentration is at least 80 µg/mL, whereas lethal blood caffeine concentration is unknown for children. In the present case, a 15-year-old girl took a large dose of an OTC antipyretic analgesic to commit suicide, resulting in caffeine intoxication. In this case, even though blood caffeine concentration was higher than the adult lethal dose, the patient recovered through a simple treatment with intravenous infusion of extracellular fluid.

## Introduction

Caffeine (1,3,7-trimethylxanthine) is a natural product that is commonly found in foods, beverages, and pharmaceuticals [1]. Recently, the presence of high concentrations of caffeine in energy drinks and over-the-counter (OTC) drugs has become a concern worldwide, not only for adults but also for adolescents and children [2]. Several deaths due to caffeine intoxication have been reported [3].

Caffeine intoxication is often associated with gastrointestinal symptoms such as nausea and vomiting due to adenosine receptor antagonist, sinus tachycardia and hypokalemia due to catecholamine release, and, in severe cases, seizures, ventricular fibrillation, and rhabdomyolysis [4].

Ibuprofen, a nonsteroidal drug with anti-inflammatory, antipyretic, and analgesic properties, has been a prescription drug since 1985 in Japan. Caffeine is added to enhance the effect of ibuprofen in OTC drugs [5], although the mechanism that enhances the antipyretic effects of ibuprofen with caffeine is still not fully understood.

Here, we report a case of caffeine intoxication in a 15-year-old girl who took a large amount of an OTC drug with antipyretic analgesics.

## Case presentation

The patient was a 15-year-old girl with no diseases underlying her condition. She had no prior episodes of drug overdose or history of psychiatric visits. She consumed 68 tablets of OTC antipyretic analgesics (Norshin Pure®; 75 mg ibuprofen with 40 mg caffeine per tablet) to commit suicide. The total amount of caffeine in 68 tablets was 2,720 mg. She was referred to our hospital due to recurrent vomiting and unconsciousness 11 hours after the estimated time of consuming the tablets and presented to the initial laboratory at the same time.

On admission, her Glasgow Coma Scale score was 11 (E3V3M5), her eyes opened in response to verbal stimuli, and she had purposeful motor response to painful stimuli; however, her best verbal response was inappropriate words. Her respiratory rate was 22 breaths per minute, her blood pressure was 99/66 mmHg, and her heart rate was 110 beats per minute. The patient continued to vomit repeatedly at the hospital. She was diagnosed with a drug overdose based on her symptoms and consultation with her grandmother who lived with her. Electrocardiography showed sinus tachycardia with no other abnormal findings. Chest and abdominal X-rays also showed no abnormal findings.

The patient’s laboratory data are listed in Table 1. We administered intravenous fluids (sodium 131 mEq/L, potassium 20 mEq/L, and 5% glucose) to help speed up caffeine elimination. Activated charcoal was not administered because of difficulty with gastric tube insertion due to the patient’s irritability. Serum pH, electrolytes, creatine kinase, and glucose were within normal range, and her vital signs were stable. Unconsciousness improved 17 hours after admission; however, the patient’s suicidal inclinations remained. The attending psychiatrist decided to transfer her to a psychiatric hospital on the second day of admission. Blood caffeine concentration was 71.5 µg/mL at admission and 37.4 µg/mL 17 hours after admission. Blood ibuprofen concentration was 45.3 µg/mL at admission and 0.8 µg/mL 17 hours after admission. The highest blood caffeine and ibuprofen concentrations were estimated to be 108.8 μg/mL of caffeine and 423.3 μg/mL of ibuprofen.

**Table 1:** Laboratory data on admission. WBC: white blood cell; RBC: red blood cell; BUN: blood urea nitrogen; AST: aspartate aminotransferase; ALT: alanine aminotransferase; LDH: lactate dehydrogenase; CK: creatine kinase

	Data	Reference range
pH	7.367	7.35–7.45
pCO_2 _(mmHg)	38.6	35–45
HCO_3 _(mmol/L)	21.7	23–28
Base excess (mmol/L)	-3.3	-2–+2
Lactate (mmol/L)	4.6	<1.6
WBC (/µL)	17.50×10^3^	3.3–8.6×10^3^
RBC (/µL)	445×10^4^	386–492×10^4^
Hemoglobin (g/dL)	12.8	11.6–14.8
Platelet count(/µL)	31.7×10^4^	15.8–34.8×10^4^
Glucose (mg/dL)	152	73–109
Sodium (mmol/L)	140	138–145
Potassium (mmol/L)	3.2	3.6–4.8
Chloride (mmol/L)	103	101–108
BUN (mg/dL)	7	8–20
Creatinine (mg/dL)	0.51	0.65–1.07
AST (U/L)	14	13–30
ALT (U/L)	9	10–42
LDH (U/L)	180	124–222
CK (U/L)	134	59–248

An estimated 250 mg of caffeine would result in a blood concentration of approximately 10 µg/mL according to the Japanese Pharmacopoeia. Considering that the patient took 2,720 mg of caffeine, the estimated maximum blood concentration was 108.8 μg/mL (2,720/250 × 10).

## Discussion

A lethal blood caffeine concentration, which is at least 80 µg/mL in adults [6] but unknown in children, seems to have a low correlation with clinical symptoms. The life-threatening ibuprofen concentration for children is at least 400 mg/kg but remains unknown for adults [7]. Although our patient’s blood caffeine level was over the adult lethal dose, her clinical symptoms and laboratory findings improved considerably with intravenous fluid therapy, deeming activated charcoal administration or hemodialysis unnecessary. Furthermore, as caffeine elimination occurs mostly through renal excretion in urine (85-88%) [4], elimination was expected by intravenous fluid therapy. Active charcoal was not administered due to the irritability of the patient. In a similar report of a 14-year-old girl who overdosed on caffeine to commit suicide, the treatment was only with intravenous infusion, and no complications arose, although her blood caffeine concentration was 198 μg/mL [8].

Caffeine generally reaches peak plasma concentrations within 30-120 minutes after administration, and the elimination half-life is an average of approximately three to six hours in healthy humans [4]. However, it has been reported that the elimination half-life can be extended up to 15 hours in cases of overdose [9]. If the blood concentration at 11 hours after the estimated time of administration was 71.5 µg/mL, the estimated maximum blood concentration would have been at least 108.8 µg/mL.

Lethal caffeine concentrations may be higher in children than in adults. This difference may be explained by the mechanisms involved in caffeine metabolism such as CYP1A2 activity. CYP1A2 activity depends on genetic factors, suggesting individual differences in caffeine overdose thresholds. Factors such as pregnancy, female gender with liver disease, grapefruit juice consumption, contraceptive use, and alcohol consumption also reduce CYP1A2 activity [4]. Caffeine clearance appears to vary with age. In a caffeine breath test, children aged three to nine years had 50% higher caffeine clearance than adults, whereas children aged 9-15 years had 33% higher caffeine clearance than adults [10]. Similar to our case, Noto et al. [8] reported a case with caffeine concentrations higher than the adult lethal dose and was resolved without complications, possibly because CYP1A2 activity in adolescents differs from that in adults. Nevertheless, further case studies are required to make conclusive evaluations of the lethal caffeine concentration thresholds in children and adolescents.

Previously reported cases of caffeine intoxication in adolescents are summarized in Table 2. Several deaths due to caffeine intoxication have been reported. In our patient, the amount of caffeine consumed was low compared to other reported cases. Although there appears to be a trend of higher mortality with higher caffeine intake, the cutoff concentration is not known. There have been several cases where the adult lethal blood concentration of 80 µg/mL was exceeded but the patient recovered. The current case is the second youngest after the case reported by Noto et al. [8] and may be difficult to compare with other cases in the literature. As the number of reports of caffeine intoxication in adolescents is small, further exploration is necessary. Children and adolescents with stable clinical and laboratory findings and caffeine concentrations greater than 80 µg/mL may improve by simple treatments such as intravenous infusion. The lethal level of 80 µg/mL described in adults may be higher in children and adolescents which needs to be further explored.

**Table 2:** Previously reported cases of caffeine intoxication in adolescents.

Case [Reference number]	Age	Gender	Origin	Total caffeine dose	Symptoms	Blood caffeine concentration (µg/mL)	Treatment	Outcome
This case	15	F	Over-the-counter drugs	2.72 g	Unconsciousness	108.8	Intravenous infusion	Recovered
Yamamoto et al. [3]	18	F	Over-the-counter sleep inhibitor product	51.6 g	Unknown	290	Not performed	Died
Noto et al. [8]	14	F	Over-the-counter drugs	12.4 g	Unconsciousness	198	Intravenous infusion	Recovered
Leson et al. [9]	16	M	Tablets	6–8 g	Vomiting	91	Activated charcoal	Recovered
Babu et al. [11]	15	M	Energy drink	495 mg	Seizure	99	Unknown	Recovered
Cappelletti et al. [12]	15	F	Unknown	Unknown	Unknown	1,080	Unknown	Died
Cappelletti et al. [12]	17	Unknown	Unknown	Unknown	Unknown	Unknown	Unknown	Died
Cappelletti et al. [12]	18	F	Unknown	Unknown	Unknown	180	Unknown	Died
Cappelletti et al. [12]	18	M	Caffeine powder	Unknown	Unknown	>70	Unknown	Died
Usman et al. [13]	16	M	Energy drink	480 mg	Palpitation	Unknown	Medication	Recovered
Schmidt and Karlson-Stiber [14]	17	F	Caffeine tablets	12 g	Vomiting	97	Activated charcoal	Recovered
Wilson et al. [15]	17	M	Caffeinated beverages	560–800 mg	Chest pain	Unknown	Medication	Recovered
Kapur and Smith [16]	18	M	Caffeine tablets	10 g	Cardiac arrhythmia	72.5	Hemodialysis	Recovered

## Conclusions

The concentrations of lethal caffeine in the blood may be different in adults and children. Patients with concentrations above 80 µg/mL, the lethal concentration in adults, may be treated using simple treatments, such as intravenous infusion, considering the total intake, clinical symptoms, and laboratory findings; however, further case studies are required.

## References

[1] Nawrot P, Jordan S, Eastwood J, Rotstein J, Hugenholtz A, Feeley M (2003). Effects of caffeine on human health. Food Addit Contam.

[2] Wikoff D, Welsh BT, Henderson R (2017). Systematic review of the potential adverse effects of caffeine consumption in healthy adults, pregnant women, adolescents, and children. Food Chem Toxicol.

[3] Yamamoto T, Yoshizawa K, Kubo S (2015). Autopsy report for a caffeine intoxication case and review of the current literature. J Toxicol Pathol.

[4] Willson C (2018). The clinical toxicology of caffeine: a review and case study. Toxicol Rep.

[5] Moore RA, Wiffen PJ, Derry S, Maguire T, Roy YM, Tyrrell L (2015). Non-prescription (OTC) oral analgesics for acute pain - an overview of Cochrane reviews. Cochrane Database Syst Rev.

[6] Schulz M, Iwersen-Bergmann S, Andresen H, Schmoldt A (2012). Therapeutic and toxic blood concentrations of nearly 1,000 drugs and other xenobiotics. Crit Care.

[7] Volans G, Monaghan J, Colbridge M (2003). Ibuprofen overdose. Int J Clin Pract Suppl.

[8] Noto T, Hanazawa T, Yoshizawa T, Murabayashi M, Morioka I (2019). Attempted suicide by caffeine tablet overdose in a 14-year-old girl. Pediatr Int.

[9] Leson CL, McGuigan MA, Bryson SM (1988). Caffeine overdose in an adolescent male. J Toxicol Clin Toxicol.

[10] Lambert GH, Schoeller DA, Kotake AN, Flores C, Hay D (1986). The effect of age, gender, and sexual maturation on the caffeine breath test. Dev Pharmacol Ther.

[11] Babu KM, Zuckerman MD, Cherkes JK, Hack JB (2011). First-onset seizure after use of an energy drink [corrected]. Pediatr Emerg Care.

[12] Cappelletti S, Piacentino D, Fineschi V, Frati P, Cipolloni L, Aromatario M (2018). Caffeine-related deaths: manner of deaths and categories at risk. Nutrients.

[13] Usman A, Jawaid A (2012). Hypertension in a young boy: an energy drink effect. BMC Res Notes.

[14] Schmidt A, Karlson-Stiber C (2008). Caffeine poisoning and lactate rise: an overlooked toxic effect?. Acta Anaesthesiol Scand.

[15] Wilson RE, Kado HS, Samson R, Miller AB (2012). A case of caffeine-induced coronary artery vasospasm of a 17-year-old male. Cardiovasc Toxicol.

[16] Kapur R, Smith MD (2009). Treatment of cardiovascular collapse from caffeine overdose with lidocaine, phenylephrine, and hemodialysis. Am J Emerg Med.

